# Exploring ethical issues arising from a problem-solving intervention in the Swedish Primary Care

**DOI:** 10.1093/eurpub/ckac130.210

**Published:** 2022-10-25

**Authors:** I Karlsson, L Sandman, I Axén, G Bergström, L Kwak, E Sernbo, E Björk Brämberg

**Affiliations:** 1 Department of Environmental Medicine, Karolinska Institutet, Stockholm, Sweden; 2 Division of Society and Health, Linköping University, Linköping, Sweden; 3 Department of Occupational Health Sciences, University of Gävle, Gävle, Sweden; 4 Department of Social Work, Gothenburg University, Gothenburg, Sweden

## Abstract

**Background:**

Common mental disorders count for a large percentage of sick leave cases in Europe and in Sweden. Problem-solving with workplace involvement have shown promising results in reducing the number of sick leave days for employees on sick leave for these conditions. Engaging the workplace by for example including the first-line manager in the return-to-work process changes the usual role of the primary care. Hence, this study aims to explore ethical issues that potentially arise when introducing workplace involvement as part of a problem-solving intervention.

**Methods:**

A qualitative study in the Swedish Primary Care using data from semi-structured interviews with rehabilitation coordinators (n = 6), employees on sick leave for common mental disorders (n = 13), and their first-line managers (n = 8). A theoretical framework for systematic identification of ethical aspects of healthcare technologies was used to guide the interviews and reporting of results. Content analysis was used to code the data, searching for latent content. Ethical issues related to the ethical values privacy, identity, autonomy, professional values, third party, equality and justice were identified and described. The analysis was concluded by a normative discussion.

**Results:**

Ethical issues were identified such as difficulties for the employees to control personal information. A need to create an integrated role of a patient and an employee and for coordinators to act neutral instead of as a patient advocate. Managers needed to balance the needs of the organization with the needs of the employee. A pre-requisite for participation was agreeing to manager involvement which may affect the equality of the intervention.

**Conclusions:**

A conversation about sharing of information, roles, responsibilities and expectations during the rehabilitation should be initiated early and be continuous. Managers need support in learning the “how to” when having an employee on sick leave due to a common mental disorder.

**Key messages:**

- The problem-solving intervention imposed ethical issues in regard to control over personal information and role shifting, which can be handled through discussions and awareness.
- By analysing ethical aspects and norms and values connected to the intervention, adaptations and solutions can be discussed and handled before full scale implementation.

